# Approaching the SDG targets with sustained political commitment: drivers of the notable decline in maternal and neonatal mortality in Morocco

**DOI:** 10.1136/bmjgh-2022-011278

**Published:** 2024-05-06

**Authors:** Sanae Elomrani, Rachid Bezad, Vincent De Brouwere, Oona Maeve Renee Campbell, Isabelle L Lange, William Edward Oswald, Bouchra Assarag

**Affiliations:** 1 National School of Public Health, Rabat, Morocco; 2 University Mohammed V, Rabat, Morocco; 3 Public Health, Institute of Tropical Medicine, Antwerpen, Belgium; 4 Infectious Disease Epidemiology, Faculty of Epidemiology and Population Health, London School of Hygiene and Tropical Medicine, London, UK; 5 Department of Neurology, Center for Global Health, Klinikum Rechts der Isar,Technical University, Munich, Germany; 6 Global Health Division, RTI International, Research Triangle Park, North Carolina, USA

**Keywords:** maternal health, health systems, child health, qualitative study, other study design

## Abstract

**Background:**

Between 2000 and 2017/2018, Morocco reduced its maternal mortality ratio by 68% and its neonatal mortality rate by 52%—a higher improvement than other North African countries. We conducted the Exemplars in Maternal and Neonatal Health study to systematically and comprehensively research factors associated with this rapid reduction in mortality over the past two decades.

**Methods:**

The study was conducted from September 2020 to December 2021 using mixed methods, including: literature, database and document reviews, quantitative analyses of national data sets and qualitative key-informant interviews at national and district levels. Analyses were based on a conceptual framework of drivers of health and survival of mothers and neonates.

**Results:**

A favourable political and economic environment, and a high political commitment encouraged prioritisation of maternal and neonatal health (MNH) by aligning evidence-based policy and technical approaches. Five main factors accounted for Morocco’s success: (1) continuous increases in antenatal care and institutional delivery and reductions socioeconomically-based inequalities in MNH service usage; (2) health-system strengthening by expanding the network of health facilities, with increased uptake of facility birthing, scale-up of the production of midwives, reductions in financial barriers and, later in the process, attention to improving the quality of care; (3) improved underlying health status of women and changes in reproductive patterns; (4) a supportive policy and infrastructure environment; and 5) increased education and autonomy of women.

**Conclusion:**

Our study provides evidence that supportive changes in Morocco’s policy environment for maternal health, backed by greater political will and increased resources, significantly contributed to the dramatic progress in reducing maternal and neonatal mortality. While these efforts were successful in improving MNH in Morocco, several implementation challenges still require special attention and renewed political attention is needed.

**WHAT IS ALREADY KNOWN ON THIS TOPIC**

- Morocco has made outstanding progress reducing maternal and neonatal mortality over the past two decades, but the drivers of this success are not well understood.

**WHAT THIS STUDY ADDS**

- A comprehensive understanding of the drivers that led to the sharp decrease in maternal and neonatal mortality in Morocco. These include sustained political commitment for maternal and neonatal health and strengthening the translation of proposed strategies/Road Map objectives and targets into concrete actions at all levels to effectively reach all beneficiaries. The health system changes were supported by an enabling political and socioeconomic environment for women’s health and human rights.

**HOW THIS STUDY MIGHT AFFECT RESEARCH, PRACTICE OR POLICY**

- In highlighting the drivers, especially the importance of the political ones, this research may encourage policymakers to once again put maternal and neonatal mortality reduction strategies higher on the political and policy agenda.

## Introduction

 Morocco is a constitutional monarchy with an elected parliament, located in North Africa, with a population of 36.9 million people in 2020, 62% of whom live in urban areas. Political stability has enabled important economic, social and institutional reforms to be undertaken,^1 2^ and the country has transitioned from being low to lower-middle-income: Morocco’s gross domestic product grew from US$3579 per capita in 2000 to US$7296 in 2020. It’s economic growth rates are still marked by significant inequalities between the poorest and richest populations and between urban and rural areas, but the distribution of national wealth has continued to be similar over time—with the Gini coefficient staying at around 40 between 1990 and 2013.

The 2011 revision of the Constitution provided a focus on health, with particular attention to rights and equity in healthcare provision. Alongside changes in the status of women, numerous policies and laws were instituted to protect women’s rights and position in the home and workplace. Free public education was extended to cover pre-primary education, attained high coverage in primary schools and progressed significantly in secondary and higher education, with gender parity in all stages.^3^

Morocco made impressive progress in improving maternal and neonatal health (MNH) outcomes over the past two decades. The maternal mortality ratio (MMR), which stood at 227 maternal deaths per 100 000 live births in 2000 decreased to 73 deaths per 100 000 live births in 2017, a decrease of nearly 68% in 20 years^4 5^ and the neonatal mortality rate (NMR) decreased by nearly 52% over the same period, from 27 deaths per 1000 live births (LB) in 2000 to 13.6 per 1000 LB in 2018.^4 5^ The MMR and NMR average annual reduction rates were higher than those of the North Africa region and of low-income and middle-income countries, after adjusting for economic development. This progress underpinned the selection of Morocco as an example of a country that made greater than average progress in reducing NMR and MMR, net of economic growth.

The Exemplars study aimed to document factors associated with reductions in maternal and neonatal mortality (MNM) over the past two decades in seven countries that have experienced greater declines than countries with similar socioeconomic progress. Understanding the reasons for a country’s success in improving MNH and reducing mortality could provide valuable guidance for maintaining course, while offering evidence and lessons to other countries embarking on a similar trajectory. We conducted an in-depth mixed-method study to uncover how Morocco achieved its progress and to identify the driving factors that brought about change. We developed and used a holistic conceptual framework for MNH research reflecting a range of inter-related factors leading to improved MNH and survival ^6^. These factors included distal determinants, such as socioeconomic, epidemiological, demographic and health systems contexts, as well as the policies and programmes deployed, mechanisms and strategies or levers used to increase the coverage and quality of high-impact MNH interventions, and to improve the preventive and positive behaviours that led to the survival gains achieved.^6^

## Methods

### Literature review

We reviewed the published scientific literature and grey literature and documents of the ministries and institutions within and outside the health sector. We developed a detailed policy timeline, and used this to analyse laws, policies, regulations and health system strategies and implementation processes that the country adopted to reduce MNM and improve MNH services.

We searched the PubMed electronic database for relevant scientific articles in French and English between 1 January 2000 and 31 December 2020. We placed no methodological restrictions and initially used broad MeSH (Medical Subject Headings) terms and keywords related to MMR, NMR, MNH determinants, strategies and interventions in Morocco. Later, we included additional terms to capture reproductive health programmes, family planning, breast feeding and women’s empowerment and financing of maternal and neonatal health programmes. After screening the titles and abstracts of 21 400 citations obtained 200 full papers were selected, read and used to inform the results.

### Secondary data analysis

Data from the Demographic and Health Survey (DHS) and other nationally representative household surveys, and Health Management Information System (HMIS) data, were also used to explore trends in contacts with antenatal care (ANC), childbirth and postnatal care services and the content of this care—and the degree that these were equitable between socioeconomic groups and geographical regions. Unfortunately, data for 2010 could not be shown as many figures were not available; 1990 data were added to complement and better show the trend.

For MMR and NMR estimates, and the causes of death we used national data from the 2004, 2011 and 2018 National Population and Family Health Surveys (ENPSF) and Maternal Deaths Surveillance System (MDSS) reports, together with modelled estimates (namely the WHO/UNFPA/UNICEF/World Bank estimates^7^) when national data were unavailable.

A decomposition method by Jain was used to assess the contribution of the fertility decline and of the safe motherhood programme to the overall decline in the MMR and NMR observed over the last two decades in Morocco.^8^

### Qualitative methods and policy review

The participants in the study was based on the actors who have contributed to the different major stages of the health-system development and/or the implementation of maternal and neonatal health policies. This pool of actors ranged from ministers to general secretaries to managers and health professionals in charge of these programmes over the past two decades. Twenty-three key informants at various levels of decision-making and implementation participated in semi-structured interviews led by a female social scientist between February and November 2021 (table 1). We developed three semi-structured interview guides adapted to different categories of actors: policymakers, MNH experts, healthcare professionals and representatives of non-healthcare institutions and partners. The main objective of these specific interview guides was to help determine the roles and perceptions of each key informant on the evolution of maternal and neonatal health policies, while considering their areas of expertise. Interviews were transcribed and coded manually. A thematic analysis was applied to analyse the qualitative data, guided by the objectives and framework of the study.

**Table 1:** Key informant affiliation

Institution	Profile of actors	Number
Ministry of health and other ministries	Policymakers	04
	National health programmes managers	02
	Directorates and social agencies involved	02
	Managers at regional health directorate level	02
	Health professionals at the level of a maternal and neonatal health facility	04
	National health insurance agency	01
Private sector	Sector representative	01
United Nations agencies	Representative per partner	02
Non-governmental organisations	Association representatives	02
Experts in maternal and newborn health	Maternal health expert	01
	Neonatal and child health expert	01
	Non-health expert (health econometrics)	01
Total		23

We integrated the quantitative and qualitative findings using the conceptual framework developed by the wider consortium for the Exemplars study,^6^ considering the observed impact on mortality, then addressing the proximate factors (intervention coverage and equity), the intermediate factors (programme and services levers and household and individual context) and the distal factors (policy and systems levers) that made proximate and intermediate factors work.

### Patient and public involvement statement

Patients and the public were not involved in this study.

A reflexivity statement is published as an online supplemental appendix 1.

## Results

The urban population steadily increased from 55% of the total population in 2004, to 60% in 2014 and to 62% in 2018,^2 9^ due mainly to rural emigration (table 2). Increased urbanisation facilitates increased geographical access to health facilities and can influence norms around fertility and women’s status, and place of birth. The performance of the Moroccan economy strengthened in the early 2000s (table 2).

**Table 2:** Changes in selected demographic, social and economic indicators in Morocco over three decades

Indicator	1990	2000	2020
Population	24 807 000	28 794 000	36 911 000
Annual population growth	1.8%	1.2%	1.2%
Population density (people per sq. km of land area)	56	65	81 (2018)
Living in urban areas (% of population)	48	53	62 (2017)
Girls married by age 15 (among women aged 20–24 and among those aged 15–49 for 2018)(%)	6.5 (1987)	2.5 (2003)	1.7 (2018)
Total fertility rate (children per woman over her lifetime)	4.0	2.8	2.4 (2019)
Annual births (thousands)*	725	640	675
Births to women aged 15–19 (per 1000)	45	35	31
Life expectancy (years)	64.7	68.7	76.7 (2019)
Under five mortality (per 1000 live births)	79	49	21 (2019)
Maternal mortality ratio (per 100 000 live births) (UN)	317	188	70
Neonatal mortality rate (per 1000 live births)	37	28	14
Mobile phone subscriptions (per 100 people)	0	8	134
Internet users, total (% of the population)	0	0.7	84
Rural population with access to electricity (UNDP)(% of population)	14 (1992)	37	99 (2019)
Population using safely managed drinking-water services (%) (UNDP)	–	55 (2000)	72
Sanitation (%)	–	35.6	47.9
Poverty headcount ratio at US $1.90 a day (2011 PPP) (% of the population)	2.9	5.8	0.9 (2013)
GDP per capita, PPP (current international $)	2549	3579	7296
Gini coefficient (World Bank)	39.2	40.6	39.5 (2013)
Human Development Index	0.457	0.529	0.686 (2019)
Female labour force participation (% of female population ages 15+) (World Bank modelled ILO estimate)	23.5	27	24 (2019)
Gender Development Index (UNDP)	0.741 (1995)	0.775	0.935 (2019)
Gender Inequality Index (UNDP)	0.722 (1995)	0.700	0.454
Literacy: male (age 15+) (proportion)	55 (1994)	66 (2004)	83 (2018)
Literacy: female (age 15+) (proportion)	28 (1994)	40 (2004)	65 (2018)
Women age 25+ years with secondary+ education (%)	9	15	29 (2019)

* See https://population.un.org/wpp/DataQuery.

GDP, gross domestic product; ILO, International Labour Organization; PPP, purchasing power parity; UN, United Nations; UNDP, United Nations Development Programme.

### Mortality and causes of death

In 1990, the MMR was 332 per 100 000 LB^10^ but witnessed a notable decrease in the years that followed. The MMR, which stood at 227 maternal deaths per 100 000 lLB in 2000,^11^ decreased by nearly 68% in 20 years, to 73 deaths per 100 000 LB in 2017.^5^

MMR declined faster than NMR (average annual rate of reduction 5.8% and 3.7%, respectively). Morocco’s NMR was around 50 per 1000 LB in the 1970s, declining to 27 per 1000 LB in 2000, 21 per 1000 LB in 2010 and 14 per 1000 LB in 2018. The NMR decreased by nearly 52% between 2000 and 2018. The share of neonatal mortality among infant deaths has increased from 57% in 1990, to 65% in 2000, to 73% in 2010 and 74% in 2018.^5^ Stillbirth rates gradually declined from 21 per 1000 total births in 2000 to 14 per 1000 total births in 2019 in public health facilities, according to annual health facility reports.^12 13^

The national Maternal Deaths Surveillance Sytem (MDSS), implemented in 2009 to analyse and understand the causes and circumstances of maternal deaths,^14^ reported data in 2010 and 2015,^15^,^17^ which indicated that specific causes of maternal deaths such as eclampsia/pre-eclampsia, complications of abortion and infection decreased by 13.5%, 59.2% and 32.2%, respectively, suggesting that the direct causes of mortality were being more effectively tackled (in relative terms) than indirect ones, except for haemorrhage (+7.7%), for which timely and high-quality care remains essential.

Unfortunately, very few data by cause of neonatal mortality are available at national level. Morocco’s national cause-specific NMR between 2000 and 2017, provided by the WHO/Maternal Child Epidemiology Estimation Group (MCEE), indicated that major reductions occurred for prematurity and for asphyxia/birth trauma and infections (intrapartum-related causes potentially addressed by better childbirth and neonatal services).

Mortality rates in rural areas remain consistently higher than in urban areas, that is, 186/100 000 LB in urban areas versus 267/100 000 LB in rural areas in 1995–2003; 73/100 000 LB in urban areas versus 148/100 000 LB in rural areas in 2009–2010 and 45/100 000 LB in urban areas versus 111/100 000LB in rural areas in 2015–2016.^4 5 11^ Although disparities were reduced between 2004 and 2018, gaps persisted between urban/rural settings, regions and different socioeconomic status of the population.

### Coverage and equity of interventions across the continuum of care

**Figure 1:**
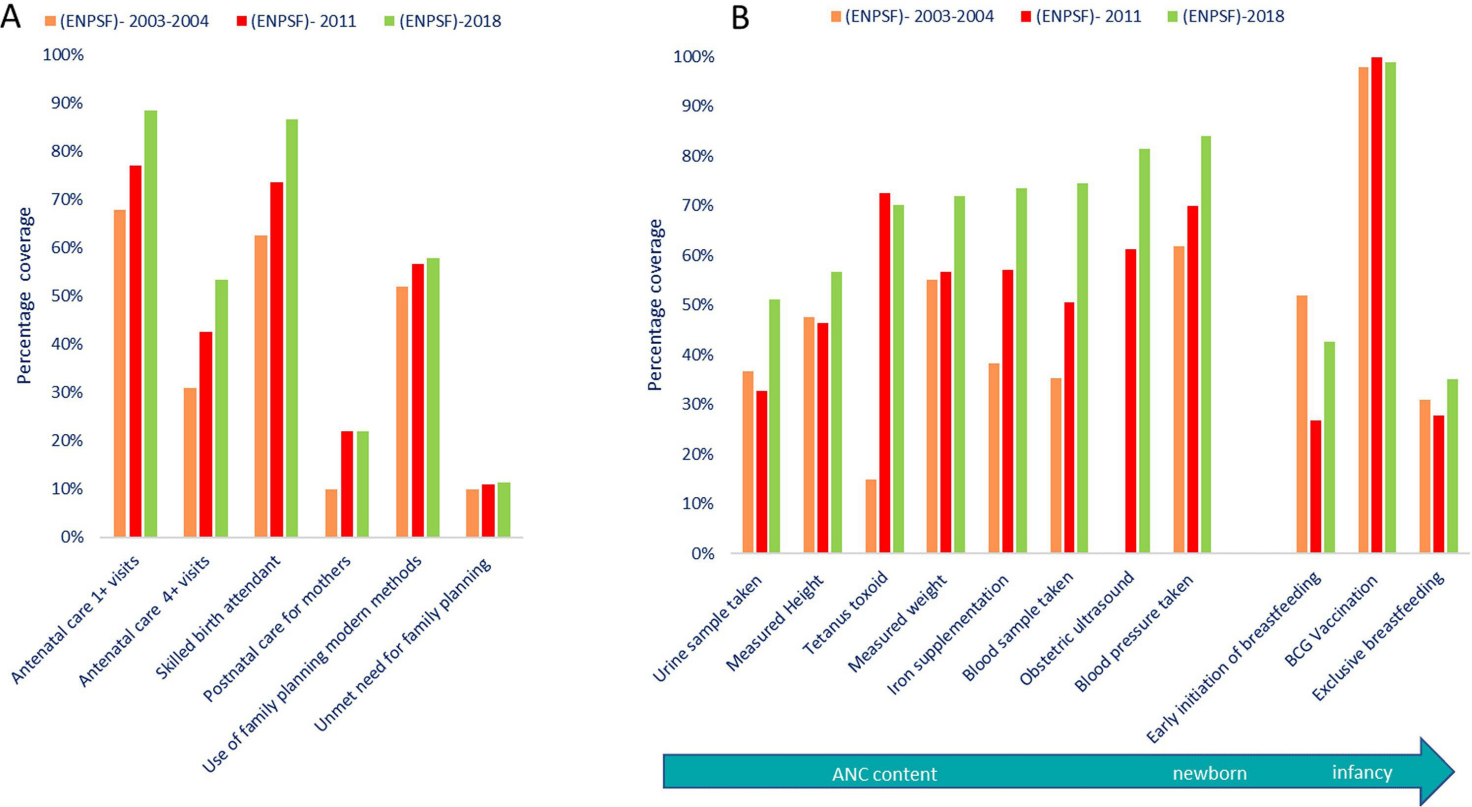
(A) Coverage of specific interventions across the continuum of care for mothers; (B) coverage of specific interventions in antenatal care and for the baby (ENPSF 2003, 2011 and 2018).

Since 2000, increasing percentages of women and neonates had contact with the health system for services in antepartum and intrapartum periods and for family planning, and to a lesser extent with postnatal services.^4 5 10 11^ The timing and number of ANC visits have also improved, as did the content of ANC and the coverage and breadth of specific interventions (figure 1).

There was a steady increase in ANC1+ coverage from 68% in 2003 to 89% by 2018.^5^ The gap between urban and rural ANC1+ coverage progressively diminished to reach a difference of 16% by 2018 (coverage of 85% in urban areas against 40% in rural areas in 1997 and 96% and 80%, respectively, in 2018). By contrast, only 54% of women had the recommended four ANC visits. Coverage of institutional deliveries reached 86% nationally in 2018 and the gap between rural and urban residence decreased over time from 53% in 1997 (81% in urban areas and 28% in rural areas) to 23% in 2018 (97% in urban areas and 74% in rural areas).

Family planning coverage (modern methods) among married women already reached 55% in 2003 and 58% by 2018.^5^ Moreover, data from the 2018 ENPSF shows that met demand is nearly at 90%.^5^ Indeed, contraception (all methods) was used by 74% of women with a secondary or higher level of education and by 70% of illiterate women.

**Figure 2:**
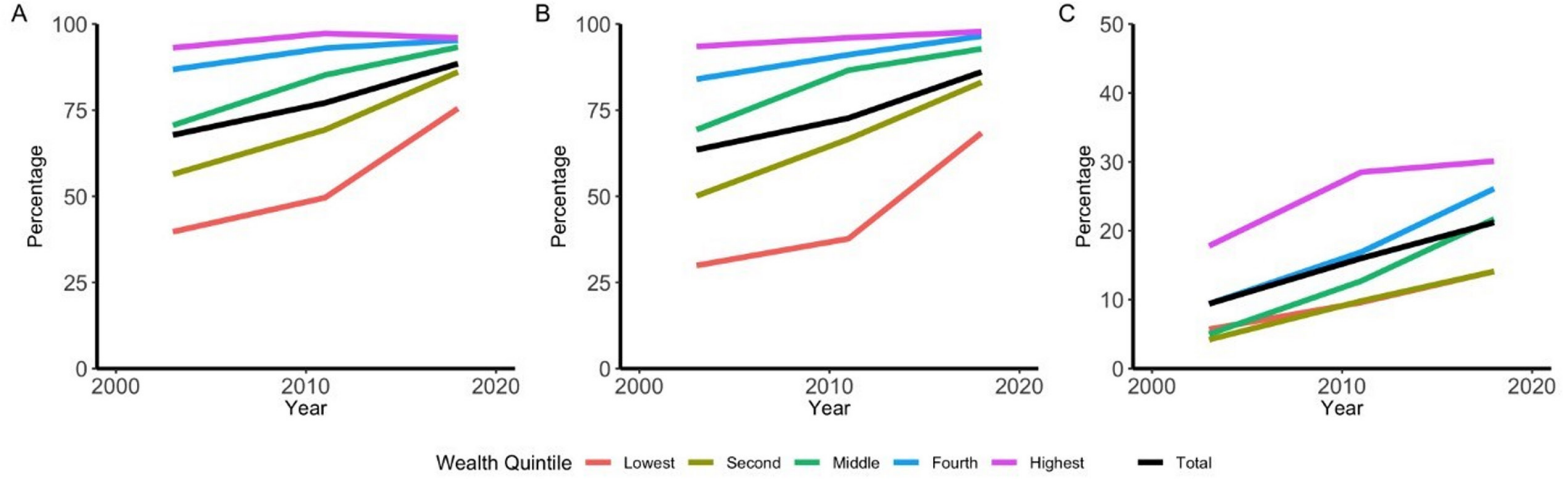
Trends in coverage of maternal health contacts: (A) Qualified antenatal care; (**B**) health facility delivery; (**C**) caesarean section by wealth quintile in Morocco.^3 4 11^

The use of caesarean section (CS) delivery increased considerably. According to household surveys, the CS rate increased from 5% in 2003 to 21% in 2018, reaching over 10% even among the poorest quintile. This national increase was driven by the increase in CSs in the private sector which reached 62% compared with 12% in public facilities.^5^ The former percentage signposted an increasing use of non-medically indicated CSs, especially among wealthier women using the private sector for delivery care (figure 2).

In general, trends in increasing contacts with services were also associated with increasing content coverage and improved quality over time (figure 1). At the same time, inequalities in coverage by rural/urban residence, regions, maternal education and household wealth showed the richer had more care, and that inequities decreased for ANC and institutional delivery over time (figure 2).

However, in 2018 the proportion of women carrying out ‘exclusive breast-feeding’ increased again (35%). The same trend was noted for the rate of early breast-feeding, which rose from 26% in 2006 to 42% in 2018 (37% in urban and 50% in rural areas).

### Household context

Incomes and household living conditions have generally improved through economic development and targeted public programmes.^1 9^ Overall, families have improved wealth, access to electricity, water and sanitary facilities and means of transport and communication, all of which are thought to have directly or indirectly contributed to reductions in mortality. For example, there have been notable infrastructural changes at the population level, with nearly 100% of households having access to electricity compared with only 70% in 2000. Internet use jumped from less than 1% in 2000 to 84% in 2020. Better road and communication infrastructure and urbanisation may have aided increased service use.^18 19^

The net enrolment rate for primary school reached 91.1% in 2017, compared with 78.6% 17 years earlier. This improvement particularly affected girls and rural dwellers, whose net enrolment rate rose between 2001 and 2017 from 75.6% to 90.9% and from 68.1% to 90.0% respectively.^20^

The time trends attest to the progress in raising education attendance at the preschool, primary school, college, qualifying and post-baccalaureate higher education levels, and in the reduction of disparities between men and women and between urban and rural areas.^21^

Women’s improved education was described by many of our key informants across different sectors as an essential element contributing to the reduction of mortality. They made links beyond simply access to information and informed decision-making:

…and then we must insist that the place of the girl is in school and university and not at home at the age of 20, it is still premature!. For example, a girl who goes to university has a high level of knowledge, she is fulfilled, she is financially independent, and she is not at the mercy of another person to buy medication, take care of her or to make a decision about treatment. MoH, national level, participant N°1

Informants suggested that thanks to the prioritisation of girls’ education, and the increase in women’s access to university, women have become more aware of the importance of monitoring their health and more autonomous in terms of access to healthcare services. The above-quoted health policy expert emphasised school as the key to health education for girls and to financial empowerment of women. However, rich-poor gaps in pre-primary education, the extent of school dropouts and dysfunctional governance mechanisms affecting the quality of learning remain powerful factors in aggravating social inequalities.^2^

Fertility patterns have changed substantially (table 2), seeing a drastic decline in women’s total fertility rate (TFR) between 1990 and 2019 from an average of four births to 2.4. In 2018, this index was 2.4 in rural areas and 2.1 in urban areas.^2 5^ We noted, however, that the TFR decreased mainly before our study period.

The fertility decline may be partly explained by the slight increased uptake of family planning services, but factors such as later marriage, the rising cost of raising children and male migrant labour trends may also have contributed to this decline. More distal drivers of these proximate determinants include increases in women’s education and status. The status of women in Morocco has undergone considerable change over the past 20 years, with increased literacy, education and greater autonomy in health and other reproductive choices. These societal advances have been reinforced by legal reforms, which have recognised and guaranteed women’s human rights (Government Plan for Equality 2012–2016 and 2017–2021).^22^,^24^ These laws and official governmental decisions are important because their existence makes it possible to refer to them to argue and defend positions in favour of health in general, and maternal and newborn health in particular.

Our decomposition analysis using the approach proposed by Jain^25^ showed that fertility contributed to saving 17% of the total number of maternal lives saved in 2017 compared with 2000, with 6.5% due to the decline in birth rates and 11% due to changing birth risk. The decomposition analysis suggests fertility effects contributed to 16% of the decline in NMR observed between 2000 and 2019. Similarly, 27% of neonatal lives were saved in 2019 due to fertility decline, compared with 2000 (14% due to decline in birth rates and 13% due to changes in birth risk composition).

Moroccan women’s underlying health status has improved, and life expectancy at birth for women has increased from 66.39 in 1987 to 77.3 years in 2014.^2^ The percentage of women with a low BMI (a risk factor for low birth weight) or with anaemia has decreased. Although these improvements may partly explain why the percentage of low birthweight babies has also decreased, there is in parallel an increasing proportion of overweight and obese women which is associated with adverse consequences for mother and baby, a worrying trend that should be addressed in the future.^5^

### Health systems, policies and programme implementation

Over the past two decades, Morocco has enjoyed political stability that is uncommon in the Arab world, where many countries including Yemen, Syria, Lebanon, Palestine, Egypt, Libya and Tunisia have experienced conflict and political turmoil. The implementation of several political, economic and social reforms led to a notable improvement in the determinants of health, and in various indicators related to women’s health, such as life expectancy at birth and the TFR (table 1).^1 2^ The health system in Morocco largely involves the public sector, represented by the services of the Ministry of Health and of the Royal Armed Forces, and the private sector, both for-profit and not-for-profit. To cope with the growing demand for health services and the resulting increased pressure on the health system, Morocco has embarked on a process of reforms related to the financing and organisation of the health sector.^26^

**Figure 3:**
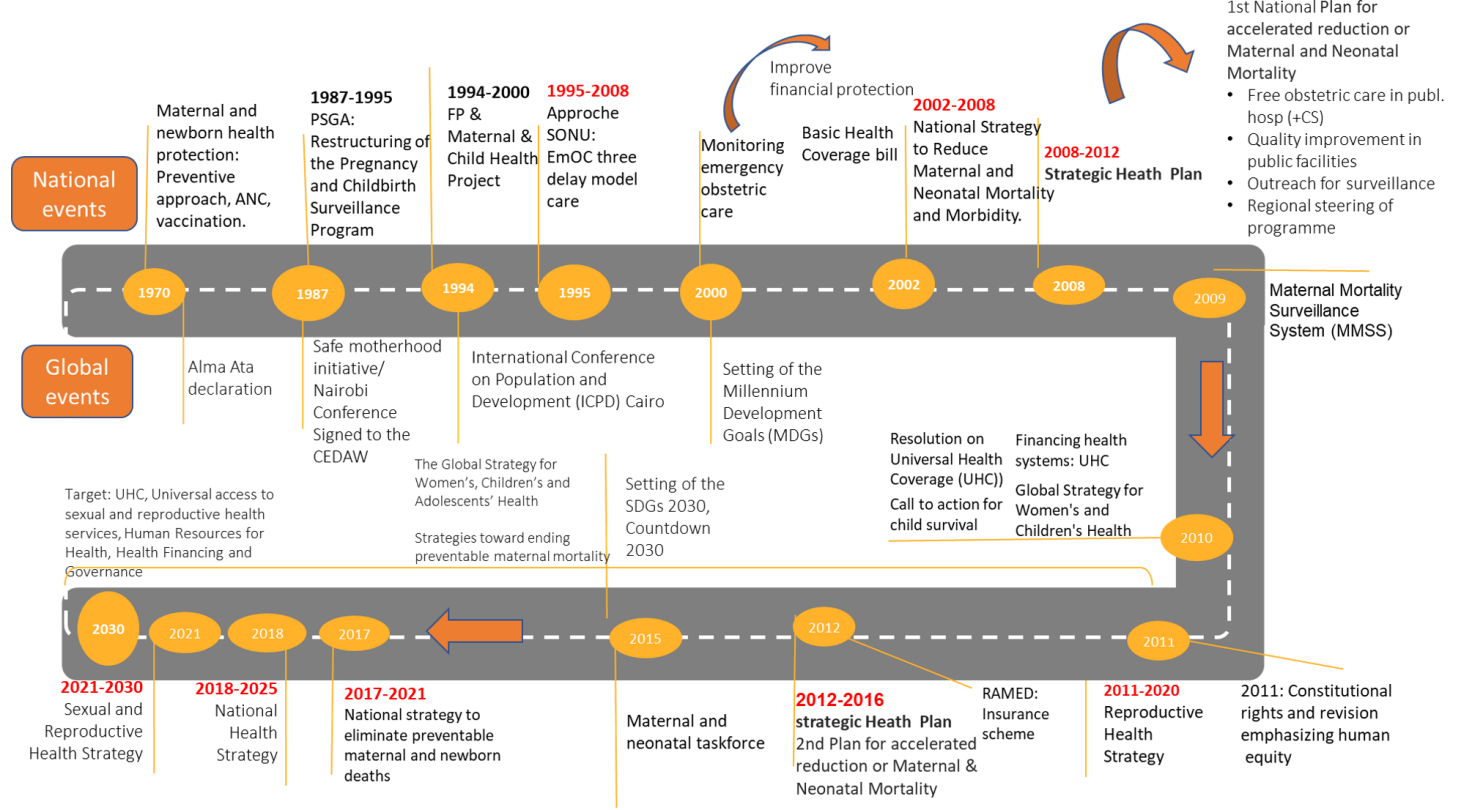
History of maternal and neonatal health policies and programmes in Morocco in light of key global events1970–2020. ANC, antenatal care; CS, caesarean section; EmOC, emergency obstetrical care; RAMED, régime d'assistance médicale – Morocco’s medical assistance scheme; SDGs, Sustainable Development Goals; CEDAW, Committee on the Elimination of Discrimination against Women; FP, Family Planning; PSGA, Programme de la Surveillance de la Grossesse et de l'Accouchement - Pregnancy and Childbirth Monitoring Program; SONU, Soins Obstétricaux et Néonataux d'Urgence - Emergency Obstetric and Neonatal Care .

Figure 3 presents the history of MNH policies and programmes in Morocco, outlining the most significant steps and the surrounding MNH events that provided the backdrop for their development. Our timeline begins some years before 2000, as earlier programmes and policies shaped the period we focused on (2000–2020) and set the scene for subsequent decisions.

Beginning in the 1980s, Moroccan programming has historically focused on the ‘risk approach’ to maternal health (meaning that women with the highest probability of suffering from pathological conditions during pregnancy or delivery are identified by a risk factor and referred to a hospital facility for special monitoring and eventual delivery in a maternity ward),^27^ which in turn benefits neonatal health. The publication of MMR data through successive demographic surveys created a national debate, especially as MMR remained high until the 2000s. Most key informants deemed that since 2008, political leadership played a significant factor in prioritising MNH and in implementing activities to accelerate the reduction of MNM, with a notable ‘champion’ of the cause found in the Minister of Health from 2008 to 2012.

We must also pay tribute to Madame Baddou. She was a female minister who gave priority to maternal health and used her political position as being from the same party as the head of government … During her mandate there was a trigger for a major investment in the fight against maternal mortality. MoH, national level, participant N°2

The action plan to accelerate the reduction of maternal mortality in 2008—2012 gave a major boost to maternal health services. With this specific national action plan, the Ministry of Health (MoH) appointed a national commission to develop a strategy and to implement the plan. Measures were the establishment of the free obstetric care policy (including newborn care), strengthening of the referral system (through ambulances, free referral between facilities levels and a specific emergency referral system in rural areas) and improved emergency care thanks to a significant increase in qualified health workers (midwives and obstetricians), equipment, drugs and consumables.^28^,^31^

Five years later, the 2012—2016 Consolidation Plan to Reduce Maternal and Neonatal Mortality was enacted to consolidate the gains from the previous years, and to reaffirm the political commitment to achieve Millennium Development Goals 4 and 5.^32^ These actions seem to have maintained the continuous decline of maternal mortality. The 2012 plan also signalled a commitment to tackling neonatal mortality in addition to maternal mortality and implementation of this policy accelerated from 2008. During the period 2008–2016 (which saw the two successive plans prioritising maternal and newborn health), decision-makers took the option of making the best use of scientific evidence and national data to make their decisions. They also involved medical professional associations, international experts, representatives of training institutions and various participants at the regional level. The secretary general and the minister regularly followed developments on the ground and used this knowledge to improve their decisions. In a way, this period saw the rise of a learning health system.

The MoH budget grew steadily, with a remarkable change in total health expenditure per capita, which tripled from US$61 to US$192 from 1997 to 2018.^33^ However, there has been only a slight change in the financing structure. Despite efforts to expand health insurance schemes, the contribution of households has remained consistently high throughout this period: 50% of financing comes from households, and the government’s share remains low. Nevertheless, a considerable portion of the government share will have gone into supporting the free obstetric care policy. The greater political will for MNH and increased resources led significantly to removing financial barriers and increasing demand for maternal health services as mentioned by several key informants.

The financial barrier has always been a big obstacle in accessing health services… The free childbirth policy which included delivery [and caesarean section], transport and referral services and care for women in maternity wards was among the main accelerators of the reduction of maternal and neonatal mortality before the RAMED (régime d'assistance médicale – Morocco’s medical assistance scheme) became more generalised. NGO president, participant N°17

In Morocco, the quantitative and qualitative deficits in human resources for healthcare are structural, and aggravated by an unbalanced regional distribution, which largely favours metropolitan regions and urban areas. Around 40% of public health professionals and 60% of healthcare professionals in both public and private sectors are concentrated in the Casablanca-Settat and Rabat-Salé-Kénitra regions where 34% of the population lives.^26^

**Figure 4:**
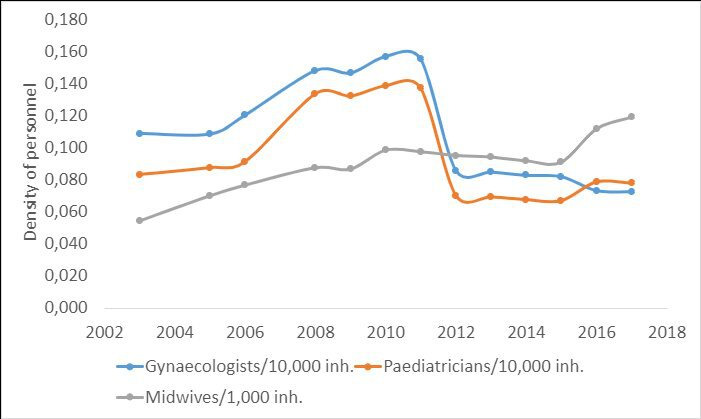
Density of workforce trends among health professionals involved in maternal and neonatal health in the public sector, 2003—2017

Within the framework of capacity building, an action plan was developed and implemented in 2009, specifically targeting the strengthening of the role of midwives. Its objective was to produce qualified midwives who could actively contribute to achieving the national strategy’s goals.^34^ As such, the public sector saw a marked increase in the number of active midwives between 2003 and 2010, a stagnation between 2011 and 2015 and a continuous increase from 2015 to 2020 (figure 4), in line with the increase in institutional deliveries during the same period.

During this period, the density of public-sector obstetrician gynaecologists and paediatricians dramatically increased between 2008 and 2011. This was the result of the political will to successfully force obstetrician and paediatrician students to practise their training period in rural hospitals. Thereafter, the density fell sharply in 2012 to a level that did not match that before Health Minister Baddou’s policy in 2008. This was the result of obstetricians and paediatricians moving from the public sector to the private one because employment conditions in the public sector did not meet their expectations and subsequent ministers were unable to force students to work in remote areas.

In 2011, the MoH implemented a ‘Regional Scheme of Health Services Organisation’ policy to strengthen the network of health facilities in terms of staffing, equipment, number of facilities and equitable implementation of programmes.^26 35^

The availability of primary healthcare has remained constant since the 1990s, while the availability of hospitals has improved, particularly after the 2012–2016 MoH consolidated plan. Inequalities in access, suboptimal quality and population’s rising expectations remain the main challenges.^12 26^ The big increase in institutional delivery was in childbirth at the hospital level, not lower-level facilities.^12^ The availability of basic and comprehensive emergency obstetrical and neonatal care has increased, especially since 2008.^36^ We also note that more than 80% of institutional deliveries took place in hospitals.^12^ Indeed, skilled birth attendance increased from 31% in 1992 to 48.4% in 1997, 62.6% in 2004 and 73.6% in 2011 to 86.6% in 2018. The gap between urban and rural areas narrowed from 45.8% in 2004 to 37.1% in 2011 and 22.4% in 2018.

## Discussion

Findings regarding health service and programme levers support that health systems interventions are key in reducing maternal and neonatal mortality.^37^ The reduction of maternal and neonatal mortality is a clearly stated priority of the government of the Kingdom of Morocco. The emergence of maternal health as a top priority policy is associated with the political leadership of the first female health minister, in 2008–2012. Maternal mortality became a hot debate topic at the national and subnational levels. Policymakers played a critical role in the agenda setting in health matters.^30 38 39^ We argue that this minister’s leadership triggered the activities which contributed to the acceleration of the decline in maternal and neonatal mortality. Scholars who analyse how certain health issues achieve prioritisation and allocation of resources, have found that alignment with national or global norms are critical to receiving increased attention,^40^ and the run-up to the deadline of the Millennium Development Goals (in 2015) provided the global backdrop for increased action to meet the targets. Amplified concern about the status of women, advocacy on the part of women’s rights activists and the aforementioned social and constitutional reforms demonstrate that the landscape in Morocco may have been primed for a focus on maternal and neonatal health. Indeed, during Minister Baddou’s tenure, additional efforts were made to strengthen the maternal and neonatal health service package of interventions that included ANC, institutional delivery, immediate postpartum surveillance, a strengthened referral system targeting high-risk pregnancies, strengthened essential basic and comprehensive emergency neonatal and obstetrical care and increased building linkage between lower-level and higher-level maternal facilities and emergency medical transfers in rural areas. The free birth policy, a major decision of the minister in 2009, was the turning point in the history of maternal and neonatal health in Morocco that allowed the significant increase in equity and access to health facility delivery and CSs in Morocco.^29 41 42^ Strengthening health facilities and equipment, as well as investing in midwives, have been strategic elements contributing to the reduction of maternal and neonatal mortality. The concerted investment in midwives began in the early 2000s, followed later by the 2009 user-fee exemption policy and attention to quality of care at health facilities.^43 44^ Several studies have documented positive changes in socioeconomic inequalities in the key coverage indicators. Growth in hospital deliveries was substantial. More interestingly, the top wealth quintile makes substantial and increasing use of private deliveries while the poorest groups are closer to the 10–15% CS rate considered for medically necessary indications. Thus, the gap in CS rates between the public and private sector has widened, and the alarming rate of CS in the private sector calls for measures to regulate this practice.

In Morocco, access, availability, usage and coverage of maternal and neonatal care services have improved significantly, but the quality of this care remains an obstacle to accelerating the reduction of maternal and neonatal mortality and morbidity. The policy of free delivery largely contributed to the improvement of access to supervised delivery, with an impact on the quality of care. The quality assessment survey revealed shortcomings in terms of implementation of national policies and guidelines for quality maternal and neonatal healthcare adopted by the MoH to achieve optimal care for women and neonates.^45^ The national survey identified gaps in the quality of maternal and neonatal care at multiple levels; (1) continuity of services related to the management of health workers and the inadequacy between position and profile, and shortcomings relating to the maintenance of equipment, (2) non-compliance with protocols and recommendations for good practice and (3) problems of interpersonal communication, respect for the dignity of women and humanisation of services.^45^

Morocco has undergone major demographic and socioeconomic changes over the last three decades. The implementation of several political, economic and social reforms has led to a significant improvement in incomes and household living conditions. Alongside changes in the status of women, numerous policies and laws were instituted to protect women’s rights and increased education and women’s autonomy.

Better road and communication infrastructure and urbanisation may have aided increased service use, especially at the time of childbirth when barriers to reaching a hospital can sometimes become a life-threatening emergency. In addition, over the study period, many Moroccans saw a gradual increase in their income; low income levels being a key risk factor for maternal death and highly associated with the three delays (decision to seek care, access to care and timeliness and quality of care)^46^,^48^; financial protection was put in place specifically for delivery care and referral in 2009.

Moroccan women’s underlying health status has improved, and low birth weight has reduced. Notable declines in fertility and an increase in age at marriage, and women’s increasing access to education and improved social status are also important contributors. Fertility declines can affect the numbers of maternal deaths and the risk of maternal death in multiple ways. First, fewer births result in fewer maternal deaths. Second, the fertility decline may shift the age-parity distribution of births to favour those at reduced maternal mortality risk, since risks tend to be higher at younger maternal age^49^ or at older maternal age or higher parity. Third, couples increasingly feel the opportunity cost of having a child and raising it to the best of their ability as the family networks that used to provide care and education for children are weakening.^50^ The contribution of fertility decline was shown to be moderately important by our Jain decomposition analysis; however, most of the decline occurred before 2000.

## Conclusion

Our study provides evidence that changes in Morocco’s demographic, sociocultural and economic circumstances created a supportive policy environment for maternal health backed by greater political will and increased resources significantly which contributed to the improvement of MNH in Morocco. The learning health system that was set up during the 2008–2016 period contributed to the reduction of inequality in access to essential obstetrical and neonatal care, and improvement of the quality of the care provided. This also led to several opportunities in terms of policies, guidelines and programmes for improving MNH that contributed to the reduction in maternal and neonatal mortality. The influence of events and developments has enabled Morocco to make great strides in improving maternal and neonatal health, which has been a national priority for the past 20 years. Several challenges remain to be addressed to achieve the Sustainable Development Goals, including addressing geographical disparities and improving the quality of care and adopting a woman-centred approach to health, filling data gaps in MNH, increasing investment in human resources for MNH and adopting a systematic evaluation approach to maternal and child health initiatives and experiences. These challenges will not be addressed without a renewed commitment from the MoH and the government.

## Supplementary material

10.1136/bmjgh-2022-011278 online supplemental file 1

## Data Availability

All data relevant to the study are included in the article or uploaded as supplementary information.
