# Supplementary material for: Approaching the SDG targets with sustained political commitment: drivers of the notable decline in maternal and neonatal mortality in Morocco
Source: BMJ Glob Health. 2024 May 6;9(Suppl 2):e011278. doi: 10.1136/bmjgh-2022-011278 (PMC11085709; doi:10.1136/bmjgh-2022-011278)
Supplement: online supplemental file 1 [file bmjgh-2022-011278supp001.pdf]

Appendix: Reflexivity statement

Questions		Responses
Study conceptualisation	1 How does this study address local research and policy priorities?	Morocco made impressive progress in improving maternal and newborn health outcomes over the past two decades. How did Morocco achieve this progress and what were the driving factors? What were the obstacles? The answer to these questions will help Morocco maintain its course, while offering evidence and lessons to other countries embarking on the same journey. The opportunity to reflect on longer term progress is also useful for local researchers
	2 How were local researchers involved in study design?	Although the initial concept of the study was proposed by a consortium of Johns Hopkins, University of Manitoba and the London School of Hygiene & Tropical Medicine, local researchers (Moroccan) developed the study design collaboratively with other members of the international research team and led on data collection, analysis and write-up. We have used the appropriate approach and tools to systematically and comprehensively research and document factors associated with rapid reductions in maternal and neonatal mortality over the past two decades
Research management	3 How has funding been used to support the local research team(s)?	Funding was granted directly to the local team and was used by the local team to support the design, collection and analysis of data to complete the study. Also, to participate in the workshop related to the research project
Data acquisition and analysis	4 How are research staff who conducted data collection acknowledged?	The research staff who carried out the data collection are among the authors of the manuscript, including lead and senior authors.
	5 How have members of the research partnership been provided with access to study data?	Permission to access the data needed to conduct the study was granted to the local research team by the Moroccan Ministry of Health and Social Protection. The entire research team had access to the data needed to perform the data analysis.
	6 How were data used to develop analytical skills within the partnership?	The research experience was a learning and sharing process, all data was analysed in a collaborative manner and experiences were exchanged within the research team and teams from the other "Exemplars in Maternal and Newborn Health" countries. Researcher participated in regular international meetings (Zoom) and presented their work and ideas

Questions		Responses
Data interpretation	7 How have research partners collaborated in interpreting study data?	The whole team participated in the interpretation of the data while benefiting from the expertise of the international partners in the analysis of maternal and neonatal health data, which added value to our study.
Drafting and revising for intellectual content	8 How were research partners supported to develop writing skills?	The research team was supported throughout the research process, including writing the report and the article. It has gone through several rounds of proofreading and revisions. Critical reading of articles from other countries participating in the project contributed to improve writing skills.
	9 How will research products be shared to address local needs?	Research products were shared to meet local needs through several stakeholders' meetings and the development and sharing of a policy brief based on the study findings. Through this manuscript, we also wish to share the results with the scientific community and people interested in this field.
Authorship	10 How is the leadership, contribution and ownership of this work by LMIC researchers recognised within the authorship?	All the study team members are authors. The first and last authors of the manuscript are part of the local research team.
	11 How have early career researchers across the partnership been included within the authorship team?	The research team ensured from the start that a young researcher, preparing her doctorate, was involved throughout the process and also in the writing of the manuscript. She is the corresponding author of this article.
	12 How has gender balance been addressed within the authorship?	Gender balance has been respected within authorship; the majority of authors are female scientists.
Training	13 How has the project contributed to training of LMIC researchers?	Thanks to exchanges with international expert (meetings, email exchanges), the local team improved its skills in analysing data and writing
Infrastructure	14 How has the project contributed to improvements in local infrastructure?	Not applicable
Governance	15 What safeguarding procedures were used to protect local study participants and researchers?	There were no particular procedures, maybe because the international promoters were willing to respect their middle-income country partners
